# Implementing value-based care for outpatient conditions in an Asian public healthcare system: framework and case studies from Singapore General Hospital

**DOI:** 10.3389/fpubh.2026.1795985

**Published:** 2026-04-15

**Authors:** De Zhi Chin, Wan Jin Sia, Yang Lv, Elisabeth Angelina, Tessa Xuan Rui Teo, Hao Yi Tan, Danny Jon Nian Wong, Aaron Kian Ti Tong, Elizabeth Sein Jieh Tan, Hairil Rizal Abdullah

**Affiliations:** 1 Office of Value Based Healthcare, Singapore General Hospital, Singapore, Singapore; 2 Department of Anaesthesiology, Singapore General Hospital, Singapore, Singapore; 3 Department of Nuclear Medicine and Molecular Imaging, Singapore General Hospital, Singapore, Singapore; 4 Department of Pain Medicine, Singapore General Hospital, Singapore, Singapore

**Keywords:** appropriate and value based care, health system transformation, implementation science, population health, Singapore, value based healthcare (VBHC)

## Abstract

**Background:**

Escalating healthcare expenditure and population aging have intensified interest in value-based care (VBC), particularly within publicly funded health systems. To date, most value-based initiatives and published implementation experiences have focused on inpatient or procedural care. In contrast, outpatient and longitudinal conditions pose distinct challenges for outcome definition, data availability, and care coordination, and remain relatively under-documented, especially in Asian healthcare contexts.

**Innovation:**

This paper describes and operationalizes a locally developed implementation framework designed to support condition-based, value-driven outcomes monitoring for outpatient care.

**Context:**

The framework was developed and applied in a large tertiary public hospital in Singapore that has expanded its value-based initiatives beyond nationally mandated programmes.

**Approach:**

We outline a four-stage implementation framework comprising scoping, exploration and analysis, baseline assessment, and improvement planning. The application of this framework is illustrated through two outpatient case studies: radioiodine therapy for hyperthyroidism and chronic pain management.

**Key lessons:**

Across both case studies, the framework facilitated systematic outcome selection, structured engagement between clinicians and administrators, and identification of actionable opportunities for care improvement, despite data and measurement constraints inherent to outpatient settings.

**Implications:**

This community case study provides practical insights into operationalizing value-based care for outpatient and chronic disease pathways and offers transferable lessons for health systems seeking to extend value-based care beyond inpatient and procedural contexts.

## Introduction: the need for value based care

Singapore, like many healthcare systems worldwide is facing sustained cost pressures driven by demographic aging, rising chronic disease burden, and increasing expectations of care quality (1, 2). These trends have prompted growing interest in value-based care (VBC) (3, 4), which seeks to optimize health outcomes relative to the resources expended. In response, many health systems have introduced value-based or value-driven care models, supported by policy initiatives that emphasize outcomes measurement, efficiency, and sustainability alongside traditional activity-based metrics.

Despite this momentum, most documented VBC initiatives have focused on inpatient episodes and procedure-centric care, where clinical pathways are relatively bounded and outcomes easier to define. Outpatient and longitudinal care pathways on the other hand pose distinct challenges (5), including fragmented service delivery, extended time horizons for outcome measurement, and maturity of data infrastructure is more varied. As a result, there remains limited published guidance on how value-based principles can be operationalized in outpatient settings. This gap is particularly evident in Asian public healthcare systems, where outpatient services account for a substantial proportion of care delivery but implementation experiences remain under-represented in the literature.

Given these challenges, there is a need for practical, implementation-focused guidance that complements existing conceptual and evaluative VBC literature. Community case studies provide an opportunity to document real-world experience in designing and delivering value-based interventions, highlighting contextual considerations, implementation trade-offs, and early learning. Capturing such local experience is important for organizational learning and can inform policy development by illustrating how value-based care can be adapted beyond centrally defined or nationally mandated conditions. The aim of this paper is therefore to describe the development and application of a structured implementation framework for value-based care in outpatient settings at a large tertiary public hospital in Singapore, and to reflect on lessons from its early application through two outpatient case studies.

## Context

### Institutional setting

Singapore General Hospital (SGH) (6) is the largest acute tertiary hospital in Singapore and serves as the country's flagship public hospital. It is a 1,700-bed academic medical center that provides comprehensive specialist services across medical, surgical, and allied health disciplines. SGH operates within a regional public healthcare cluster that integrates acute hospitals, national specialty centers, community hospitals, and primary care providers, enabling continuity of care across settings (7). The institution employs more than 10,000 staff and serves over one million patients annually. In the outpatient setting, SGH recorded 724,480 specialist outpatient attendances in the most recent reporting year, reflecting the scale and complexity of ambulatory care delivery within the organization. As a tertiary referral center, SGH manages both high-volume routine outpatient conditions and complex cases requiring multidisciplinary, longitudinal care (8, 9).

Institutional leadership has played a central role in driving value-based initiatives. Senior clinical and administrative leaders have endorsed value-based care as a strategic priority, aligning it with national policy directions on healthcare sustainability and quality improvement (10). Governance structures support cross-departmental collaboration, data sharing, and iterative quality improvement, creating an enabling environment for experimentation with new approaches to outcomes measurement and care redesign beyond nationally mandated programmes.

### Value-based care in SGH

Value-based care efforts at SGH are coordinated through the Office of Value-Based Healthcare (OVBH) (11), a dedicated office established to support the systematic implementation of value-driven care initiatives across the institution. The OVBH is led by clinicians and supported by administrators with diverse backgrounds. This multidisciplinary composition reflects the operational nature of value-based care implementation, which requires both clinical insight and programme management capability.

The OVBH facilitates an annual institutional process to identify and prioritize new Value-Driven Conditions (VDCs). Clinical departments are invited to submit proposals for conditions where there is perceived opportunity to improve patient outcomes, enhance care processes, or optimize resource utilization. Proposals are assessed using a structured prioritization matrix that considers factors such as patient volume, potential impact on clinical outcomes, anticipated cost implications, and scalability across other departments or institutions within the healthcare cluster.

Shortlisted conditions are assigned to OVBH administrators, who partner with one or more clinician champions from the relevant specialties. Together, they scope the condition, define outcomes of interest, assess data availability, and support subsequent analysis and improvement planning. This organizational model allows value-based initiatives to be clinician-led while being operationally supported, and it enables the extension of value-based care concepts to conditions and settings not covered by national programmes.

### Target populations and care settings

While national value-driven care initiatives in Singapore have largely concentrated on inpatient episodes and procedural conditions (12, 13), a substantial proportion of healthcare utilization and expenditure occurs in the outpatient setting, particularly for chronic and recurrent conditions (14). Outpatient care poses distinct challenges for value-based implementation, including fragmented care pathways, extended time horizons for outcome measurement, and the need to incorporate patient-reported outcomes. To address this, OVBH has developed an implementation framework to guide initiatives, especially those focusing on outpatient and longitudinal care pathways.

Concurrently, two outpatient conditions were selected as illustrative case studies: hyperthyroidism treated with radioiodine therapy and high-risk chronic pain management. Hyperthyroidism managed with radioiodine represents a specialist outpatient condition characterized by defined treatment milestones but prolonged follow-up across departments, making it suitable for examining care coordination, treatment effectiveness, and discharge processes over time. Chronic pain management, by contrast, exemplifies a complex, longitudinal condition with heterogeneous patient needs, high risk of unplanned acute care utilization, and strong relevance for patient-reported and functional outcomes.

Together, these conditions were selected to demonstrate the applicability of the implementation framework across differing outpatient contexts. Radioiodine therapy represents a discrete therapeutic intervention followed by extended follow-up and cross-departmental care transitions, while chronic pain management reflects ongoing multidisciplinary care delivered largely within a single department. Rather than being selected based on disease burden, the cases were intended to illustrate how the framework can be applied across outpatient pathways with different care trajectories, highlighting its adaptability within a large Asian public healthcare system.

## The SGH value-based care implementation framework

### Design principles underpinning the framework

When the OVBH first started supporting VBC initiatives, there was no established institution-wide implementation methodology to guide the development of condition-based units within a hospital setting. Existing national programmes provided direction on what to measure for selected conditions, but offered limited operational guidance on how multidisciplinary teams should translate value-based concepts into practice, particularly for outpatient and longitudinal care pathways. As a result, early implementation efforts were heterogeneous and often relied heavily on individual experience and informal learning.

In response, the OVBH developed its own implementation framework, drawing implicitly on principles from implementation science and quality improvement. The framework was designed to support consistent, reproducible implementation while remaining sufficiently flexible to accommodate the diversity of clinical conditions and care settings encountered in a large tertiary hospital.

Several design principles informed its development. First, the framework prioritized usability by multidisciplinary teams, including administrators without formal clinical training. Given that a substantial proportion of OVBH administrators are non-clinicians, the framework needed to provide enough clinical scaffolding (through structured literature review, patient journey mapping, and guided outcome selection) to enable meaningful engagement with clinical teams. In practice however, administrators implementing the framework should have competencies in programme management, basic data analysis, literature review, and stakeholder engagement, enabling them to work effectively with clinician champions to translate clinical workflows into measurable outcomes and improvement opportunities. Second, the framework sought to balance conceptual rigor with operational feasibility. While aligned with core value-based care concepts, it deliberately avoided excessive methodological complexity that could impede uptake in routine practice. Finally, the framework was designed to be adaptable across different clinical conditions and outpatient settings, recognizing that value-based care implementation must be sensitive to local workflows, data availability, and disease trajectories.

### Overview of the four-stage framework

The SGH Value-Based Care Implementation Framework was developed to guide teams seeking to establish condition-based units in as systematic a manner as possible, while embedding learning from earlier implementation challenges. Rather than prescribing a rigid protocol, the framework functions as a structured guide to ensure that key considerations are addressed consistently across conditions.

A deliberate emphasis was placed on simplicity. The framework was designed to be readily understood and followed by clinician champions, many of whom lead busy clinical services and may have limited time to engage with complex implementation methodologies. This simplicity also facilitated shared understanding between clinicians and administrators, supporting more effective collaboration.

**Figure 1:**
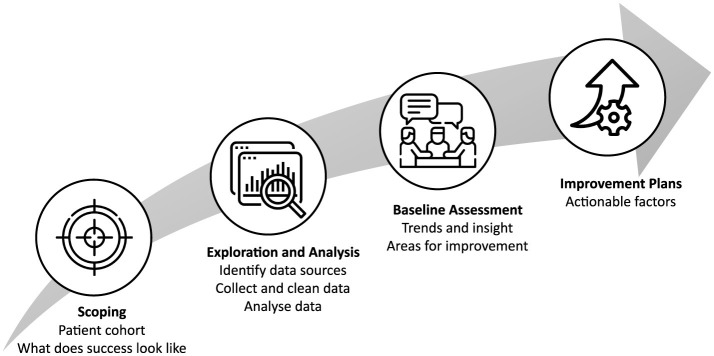
SGH's value based care implementation framework.

The framework comprises four main stages: scoping, exploration and analysis, baseline assessment, and improvement planning (Figure 1). Although presented sequentially, the stages are not strictly linear. Iteration between stages is common, particularly as new insights emerge during data exploration or baseline analysis that necessitate refinement of outcome definitions or patient cohorts. Collectively, the four stages provide a coherent structure for implementing value-based care while allowing sufficient flexibility to respond to contextual constraints and learning over time.

## Key components of the framework

### Scoping

Scoping represents the foundational stage of the framework and is critical for establishing a shared understanding of the condition and its care pathway. OVBH administrators, in collaboration with clinician champions, conduct a structured review of relevant clinical guidelines and the published literature to identify best practices, commonly reported outcomes, and potential benchmarks. This process helps situate local practice within the broader evidence base and highlights outcomes that may be meaningful from both clinical and patient perspectives.

In parallel, patient journey mapping is undertaken to visualize the full outpatient care cycle, from referral and initial assessment through treatment, follow-up, and eventual discharge or long-term management. Mapping the patient journey enables teams to identify handover points, inter-departmental dependencies, and potential sources of inefficiency or duplication.

Based on insights from the literature review and patient journey mapping, a set of outcomes spanning clinical results, process measures, and patient-reported outcomes is selected. These indicators are combined to form a condition-specific Clinical Quality Index (CQI), which represents a customized composite definition of “good care” for that condition. The CQI provides a structured way to assess performance holistically rather than focusing on single metrics in isolation. The composition of the CQI follows several guiding principles to ensure clinical relevance and interpretability. Indicators are selected through a combination of literature review, guideline recommendations, and consultation with clinician champions to identify outcomes that reflect meaningful dimensions of care for the condition. Where available, internationally recognized outcome sets (such as those developed by the International Consortium for Health Outcomes Measurement) are considered. In practice, the CQI typically combines a small number of complementary indicators spanning clinical outcomes, process measures, and patient-reported outcomes, thereby capturing different dimensions of value across the care cycle. While the exact indicators may vary across conditions and institutions due to differences in care pathways and data availability, the structured selection process helps maintain conceptual consistency while allowing pragmatic adaptation to local clinical contexts.

### Exploration and analysis

Following scoping, the feasibility of measuring the selected indicators is assessed through data exploration and analysis. This stage focuses on evaluating data availability, completeness, and reliability within existing institutional data systems. Proposed indicators are examined to determine whether they can be operationalized using routinely collected data, and whether definitions require refinement to align with real-world documentation practices.

Where ideal outcome measures are not feasible, pragmatic proxies may be considered. The use of proxies is approached cautiously and is informed by both literature review and local clinical insight. Proposed definitions and analytical approaches are shared with clinician champions, and iterative discussions are used to build confidence in data interpretation and to ensure clinical face validity. This engagement is essential for fostering trust in the data, particularly when outcomes will later be used to inform improvement initiatives.

### Baseline assessment

Once patient cohorts and outcome definitions are finalized, a baseline assessment is conducted using retrospective cohort analysis. Typically, data from the preceding 1 to 2 years are analyzed to establish baseline performance across the selected indicators. This provides an empirical understanding of current care delivery and reveals variation in performance across outcomes.

The baseline assessment allows teams to identify relatively underperforming indicators that may represent priority areas for improvement. Importantly, the intent is not to benchmark performance externally at this stage, but to develop internal insight into where care processes or outcomes may be misaligned with the intended definition of value for the condition.

### Improvement planning

The final stage of the framework focuses on translating measurement into action. Structured case reviews are conducted for patients who did not meet selected indicators, enabling teams to explore underlying reasons for underperformance. These reviews are informed by clinical records and contextual knowledge of workflows and resource constraints.

Improvement initiatives are then co-designed with clinical teams, drawing on established quality improvement methodologies within the institution. The emphasis is on identifying modifiable drivers of performance and implementing targeted changes that are feasible within existing service configurations. Improvement planning is thus grounded in empirical data, clinical insight, and operational realities, completing the cycle from outcome definition to care redesign.

Together, these four components constitute a pragmatic implementation framework that supports systematic, clinician-engaged value-based care implementation in outpatient settings. In the following sections, we illustrate the application of this framework through two outpatient case studies: radioiodine therapy for hyperthyroidism and chronic pain management. These cases were selected to demonstrate how the framework can be applied across outpatient conditions with differing clinical characteristics, care trajectories, and outcome measurement challenges, while maintaining a consistent, value-based approach to implementation.

## Case study 1: radioiodine therapy for hyperthyroidism

### Identified problem and implementation motivation

Radioiodine (RAI) therapy for hyperthyroidism is delivered predominantly as an outpatient intervention but requires prolonged follow-up and coordination across specialties, most notably between Nuclear Medicine and Endocrinology (15). While the therapeutic procedure itself is well established, post-treatment management often spans many months and involves repeated outpatient visits for biochemical monitoring, medication titration, and clinical review. This creates inherent complexity in care coordination and increases the risk of duplicated follow-up, fragmented responsibility, and inefficiencies in resource utilization.

Prior to the value-based care initiative, outcomes monitoring for RAI therapy was largely focused on procedural delivery and short-term clinical endpoints. Broader measures reflecting the full outpatient care cycle (such as timeliness of discharge from specialist follow-up, downstream healthcare utilization, and patient-reported quality of life) were not systematically assessed. This limited the ability of clinical teams to evaluate whether care was delivering value beyond technical success of the procedure. These challenges motivated the selection of RAI therapy for hyperthyroidism as an early outpatient condition for application of the SGH VBC Implementation Framework.

### Applying the framework

#### Scoping and patient journey mapping

As part of the scoping phase, OVBH administrators conducted a literature review of international and regional guidelines on the management of hyperthyroidism and radioiodine therapy, alongside published outcome measures. In parallel, the outpatient care pathway was mapped from referral through RAI administration to long-term follow-up and discharge from Nuclear Medicine. This patient journey mapping exercise clarified points of interaction between departments, timing of follow-up visits, and handover arrangements to Endocrinology.

#### Selection of outcomes

Based on the literature review and patient journey mapping, a set of outcomes spanning clinical effectiveness, process measures, and patient-reported outcomes was selected to form the condition-specific Clinical Quality Index (CQI). The CQI included indicators such as discharge from Nuclear Medicine within 365 days of RAI, absence of unplanned hospital admission within 180 days, avoidance of a second RAI dose within 365 days, and achievement of euthyroid or hypothyroid status at 6 months. To capture patient-reported quality of life, the EQ-5D-5L (16) was identified as an appropriate generic PROM for prospective collection.

#### Defining treatment success and pragmatic proxies

A key challenge arose in defining biochemical treatment success. Individual laboratory values alone were insufficient to determine whether patients had achieved euthyroid or hypothyroid status, as thyroid function tests were frequently influenced by ongoing medication titration. Through data exploration and clinician engagement, initiation of levothyroxine replacement therapy was identified as a pragmatic proxy for treatment success, as thyroxine would not be commenced if patients remained hyperthyroid. Electronic medical record review of patients who did not receive levothyroxine confirmed that a proportion had achieved euthyroid status without replacement, and this degree of imprecision was discussed and accepted by clinician champions as clinically reasonable. This process highlighted the importance of combining data analysis with clinical judgement when operationalizing outcomes.

#### Baseline insights

Baseline cohort analysis was conducted for patients who underwent RAI therapy for hyperthyroidism in 2021 and 2022, excluding those with prior radioiodine treatment. While performance was high for several clinical outcomes, discharge from Nuclear Medicine within 365 days of RAI emerged as the lowest-performing indicator. Approximately 72% of patients met this indicator in both years, indicating prolonged specialist follow-up for a substantial minority of patients.

Further examination of internal referrals revealed that a significant proportion of patients referred from Endocrinology remained under active Nuclear Medicine follow-up beyond 1 year, commonly for ongoing thyroxine dose adjustment. Additionally, overlapping appointments between Nuclear Medicine and Endocrinology clinics were observed in more than one-third of internally referred patients. These patterns suggested inefficiencies in follow-up arrangements, with implications for patient convenience, clinic capacity, and overall resource utilization.

#### Improvement actions

Improvement planning focused on redesigning follow-up workflows and clarifying inter-departmental roles in post-RAI management. Facilitated discussions between Nuclear Medicine and Endocrinology clinicians identified the scheduling of early Endocrinology follow-up as a key contributor to overlapping visits and prolonged Nuclear Medicine involvement.

Through consensus, a revised workflow was implemented in which Nuclear Medicine initiated thyroxine replacement following RAI, with subsequent dose titration transitioned to Endocrinology. Referring endocrinologists began providing open-date appointments and scheduled a six-month post-RAI Endocrinology follow-up at the point of referral. These changes were intended to streamline care, reduce duplicated appointments, and facilitate more timely discharge from Nuclear Medicine, thereby improving value by enhancing patient experience while optimizing specialist resource use.

#### Reflections

This case study highlights several important lessons for outpatient value-based care implementation. First, defining meaningful outcomes for longitudinal outpatient conditions requires moving beyond procedural success to encompass care coordination and follow-up efficiency. Second, data interpretation often necessitates the use of pragmatic proxies, and clinician engagement is essential to ensure that such proxies are clinically credible and acceptable. Finally, structured patient journey mapping and facilitated cross-specialty dialogue were critical in identifying modifiable system-level issues and co-designing feasible improvement actions. Together, these insights demonstrate how a structured framework can support outcome-driven learning and collaboration in complex outpatient care pathways.

## Case study 2: chronic pain management

### Identified problem and implementation motivation

Chronic pain management in the outpatient setting poses significant challenges due to the heterogeneous nature of pain conditions, the need for longitudinal follow-up, and the frequent involvement of multiple services over time. Within the institution, a subset of patients with chronic pain exhibited high utilization of emergency department (ED) and inpatient services, often presenting with pain-related exacerbations despite ongoing outpatient care. These unplanned episodes of care suggested gaps in symptom control, access to timely specialist review, or continuity of management.

Outpatient care pathways for chronic pain were also fragmented, with variation in referral patterns, follow-up intervals, and access to specialist appointments. Prior to the value-based care initiative, routine monitoring focused primarily on service activity rather than outcomes reflecting stability of pain control, access to care, or patient-reported health status. These factors motivated the selection of chronic pain management as a second outpatient condition to test the applicability of the SGH VBC Implementation Framework in a complex, multidisciplinary context.

### Applying the framework

#### Definition of a high-risk outpatient cohort

During the scoping phase, the clinical team and OVBH administrators agreed to focus on a high-risk subgroup of chronic pain patients who were most likely to benefit from improved outpatient management. High-risk patients were defined as those with a diagnosis of chronic pain who had at least one ED attendance or inpatient admission for pain-related issues within 6 months prior to their consultation at the Pain Management Center (PMC) (17). This operational definition aligned with the goal of reducing unplanned acute care utilization and allowed for clear cohort identification using existing administrative data.

#### Selection of outcomes

Based on evidence from the literature (18, 19) on successful chronic pain management approaches, insights from patient journey mapping, and clinical input, outcomes were selected to reflect utilization, access, and patient-centered domains. The CQI for chronic pain management included the absence of ED visits due to chronic pain within 6 months of PMC consultation, stability or lengthening of clinic revisit intervals as a proxy for symptom control, and timely access to care measured by appointment waiting times. To capture patient-reported health status, the EQ-5D-5L was selected as the preferred Patient Reported Outcome Measures (PROMs) questionnaire due to its brevity and broad applicability across conditions. At the time of baseline assessment, workflows for routine PROMs collection were still under development, and prospective implementation of EQ-5D-5L was incorporated into the improvement planning stage.

#### Baseline insights

Baseline analysis was conducted on a retrospective cohort of 727 high-risk chronic pain patients seen at the PMC in 2022 and 2023. While performance was relatively strong for indicators related to avoidance of ED visits, substantial variation was observed across other domains. In particular, timely access to care emerged as a key bottleneck. Less than half of patients met the defined appointment waiting time thresholds in both years, indicating delays in accessing specialist review despite their high-risk status.

Further examination of clinic scheduling and utilization patterns highlighted underlying workforce and operational constraints. As pain specialists are also anesthetists with operating theater responsibilities, clashes between clinic schedules and operating commitments occasionally led to underutilization of available clinic slots. These constraints limited the center's ability to offer timely appointments even when demand was evident, highlighting the importance of considering workforce deployment and scheduling practices as part of value-based outpatient care.

#### Improvement actions

Improvement planning focused on addressing access and scheduling inefficiencies identified in the baseline analysis. Proposed actions included prioritizing high-risk patients within existing appointment systems, reviewing referral histories to better anticipate demand, and expanding or reconfiguring clinic slots where feasible. These process adjustments aimed to improve timely access to care without requiring major structural changes to staffing.

In parallel, integration of PROMs into routine clinical practice was initiated as part of the value-based care implementation. The EQ-5D-5L was introduced for prospective collection to enable systematic assessment of patients' health status over time and to complement utilization and access metrics. Incorporating PROMs was intended to support more patient-centered evaluation of care and to inform future refinement of service delivery.

#### Reflections

This case study illustrates the importance of incorporating operational and access indicators when implementing value-based care for complex outpatient conditions. While traditional outcome measures often focus on clinical endpoints or acute utilization, delays in access and scheduling inefficiencies can substantially undermine the value delivered to patients with chronic pain. The structured application of the implementation framework helped surface these system-level issues and facilitated discussion of feasible improvement actions. More broadly, the case underscores the need for value-based outpatient initiatives to account for workforce configuration and operational realities alongside clinical and patient-reported outcomes.

## Cross-case insights and impact assessment

### Administrator experience with the framework

OVBH administrators reported that the implementation framework was generally easy to understand and useful in supporting their work. Responses from an anonymized internal survey of OVBH administrators were analyzed descriptively to capture perceived usability rather than to test hypotheses, and findings indicated high perceived comprehension (median 4.00, interquartile range [IQR] 3.75–4.25 on a five-point Likert scale) and practical utility (median 4.50, IQR 3.75–5.00). These perceptions were consistent with administrators' experience of using the framework to structure discussions with clinical teams, guide literature review, and translate clinical processes into measurable indicators.

However, perceptions of efficiency gains were more mixed. Respondents were more neutral when asked whether the framework improved work efficiency (median 3.50, IQR 3.25–4.25). This reflected the additional upfront effort required for systematic scoping, patient journey mapping, and data validation, particularly for outpatient conditions where outcomes and care boundaries were less clearly defined. Despite this, most respondents indicated that they would continue to use the framework in future value-driven care initiatives (median 4.00, IQR 4.00–5.00), suggesting that its perceived benefits outweighed the additional time investment.

Differences were observed between junior and senior administrators. Junior staff, who had only worked on value-driven conditions after the introduction of the framework, consistently rated ease of understanding, usefulness, and efficiency higher than senior staff. In contrast, senior staff (many of whom had prior experience implementing conditions without a formalized framework) tended to use the framework more as a reference guide rather than a strict step-by-step process. Follow-up interviews revealed that senior staff often relied on tacit knowledge developed through earlier projects, whereas junior staff benefited more directly from the explicit structure and guidance provided by the framework.

### Organizational learning

Beyond individual user experience, the framework played an important role in organizational learning and capability building. By providing a common structure for scoping, analysis, communication of findings, and improvement planning, the framework helped standardize how value-based care initiatives were discussed and developed across different clinical conditions. This consistency reduced variability in implementation approaches and facilitated clearer communication between administrators and clinician champions, particularly in multidisciplinary settings.

**Figure 2:**
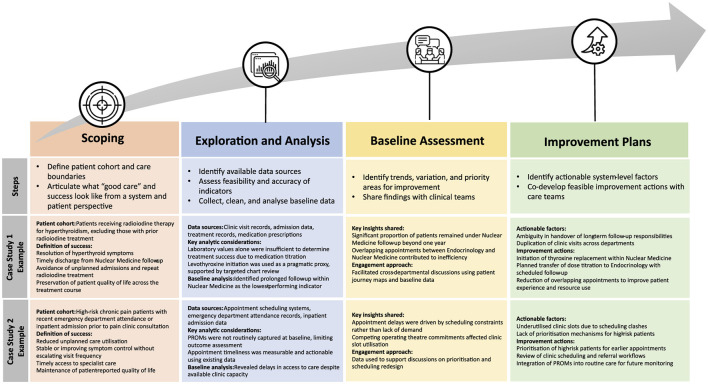
Application of the SGH value-based care implementation framework across two outpatient case studies.

The cross-case comparison of radioiodine therapy for hyperthyroidism and chronic pain management highlighted how the same framework could be applied to outpatient conditions with markedly different care trajectories. As illustrated in Figure 2 below, both cases followed the same overarching stages (i.e., scoping, exploration and analysis, communication of results, and improvement planning) while yielding condition-specific insights and actions. In the radioiodine case, the framework helped surface inefficiencies related to prolonged follow-up and overlapping clinic visits across departments, whereas in chronic pain management it highlighted access and scheduling constraints driven by workforce and operational factors. Presenting these cases side by side within a single framework reinforced shared learning across teams and demonstrated the adaptability of the approach.

At an institutional level, the framework contributed to the development of a more systematic internal capability for VBC implementation. Administrators without formal clinical training were better able to engage meaningfully with complex outpatient conditions, while clinician champions were provided with a clear structure to articulate what “good care” looked like beyond procedural outcomes. Over time, this has supported more consistent implementation practices and created a shared language for discussing outcomes, data limitations, and improvement priorities across the organization.

## Discussion: practical implications and lessons for health systems

This case study contributes to the VBC literature by extending implementation insights beyond inpatient and procedural contexts into outpatient and longitudinal care pathways. Much of the existing published experience on VBC implementation, including national programmes in Singapore, has focused on acute hospital episodes and elective surgical procedures, where care boundaries and outcomes are relatively well defined. In contrast, the outpatient conditions presented in this paper span longer time horizons, involve multiple specialties, and require consideration of process and access measures alongside traditional clinical outcomes.

By documenting how value-based principles were operationalized for outpatient settings, this study demonstrates that systematic outcomes measurement and improvement planning are feasible even when care pathways are complex and data are imperfect. The use of pragmatic proxies, patient journey mapping, and condition-specific CQIs illustrate how institutions can adapt value-based concepts to settings where traditional indicators may be insufficient. Importantly, this experience adds an Asian public healthcare system perspective to the literature, which remains under-represented despite rapid population aging and rising chronic disease burden in the region. The case studies reflect the realities of publicly funded, high-volume outpatient care and highlight implementation challenges and solutions that may differ from those reported in predominantly Western health systems.

### Implications for health economics and system sustainability

From a health economics perspective, the framework illustrates how systematic outcomes measurement can be linked to more informed resource allocation and efficiency improvement. In both case studies, baseline analysis identified system-level inefficiencies that were not readily apparent from traditional activity-based metrics alone. In the radioiodine therapy case, prolonged follow-up and overlapping clinic visits across departments suggested suboptimal use of specialist resources. In chronic pain management, delays in access to care were driven not by lack of demand but by scheduling and workforce constraints, leading to underutilization of available clinic capacity.

By making such inefficiencies visible, the framework supports targeted improvement actions that have the potential to improve value without necessarily increasing resource input. This is particularly relevant for outpatient care, where a large proportion of chronic disease management occurs and where incremental inefficiencies can cumulatively exert substantial cost pressure on health systems. Incorporating utilization, access, and patient-reported outcomes alongside clinical indicators enables a more holistic assessment of value and aligns improvement efforts with broader system sustainability goals. As health systems face increasing demand from aging populations, outpatient value-based care offers a mechanism to manage chronic disease burden more effectively by optimizing care pathways rather than expanding capacity alone.

By making such inefficiencies visible, the framework supports targeted improvement actions that have the potential to improve value without necessarily increasing resource inputs. This is particularly relevant in outpatient care, where chronic disease management accounts for a substantial proportion of healthcare utilization and expenditure, and where incremental inefficiencies can cumulatively exert significant cost pressure on health systems. Incorporating utilization, access, and patient-reported outcomes alongside clinical indicators enables a more holistic assessment of value and aligns improvement efforts with broader system sustainability goals. From a public health perspective, outpatient VBC is especially important for strengthening chronic disease management, improving access to timely specialist care, and reducing avoidable unplanned acute care utilization; key contributors to system resilience in aging populations.

### Transferability and scalability

The SGH Value-Based Care Implementation Framework was deliberately designed to be pragmatic and adaptable, making it potentially transferable to other institutions, particularly those at an early stage of VBC implementation. Key conditions for successful adoption include the presence of clinician leadership, dedicated administrative support, and access to clinical and administrative data. Importantly, the framework does not require sophisticated costing methodologies or fully mature patient-reported outcome infrastructures at the outset, allowing institutions with nascent or under-developed VBC efforts to begin implementation using available data and progressively refine indicators over time. While the presence of a dedicated organizational unit such as the OVBH can facilitate coordination, methodological support, and scaling of initiatives across departments, value-based care implementation can also begin through smaller, clinician-led or department-level efforts that adopt similar principles of outcome measurement, patient journey mapping, and iterative improvement.

The emphasis on structured scoping, patient journey mapping, and iterative engagement with clinical teams is likely to be relevant across different organizational and financing initiatives by providing operational guidance on how value-based care can be implemented beyond centrally defined conditions. The experience described in this study suggests that such frameworks can support institutional learning, enable more consistent implementation practices, and provide a foundation for scaling value-based care efforts across healthcare clusters or systems.

Although this implementation framework was developed within a large tertiary hospital with access to established data infrastructure, its core components are not inherently dependent on advanced technological capabilities. Smaller centers may operationalize the framework by adopting a more pragmatic approach to data collection and analysis, drawing on routinely available administrative data, manual chart review, or small-scale audit processes where necessary. For example, patient journey mapping and outcome selection can be conducted through multidisciplinary clinical discussions and review of local practice patterns, while baseline assessments may initially focus on a limited number of high-priority indicators that can be reliably measured with available data. As data systems mature over time, additional outcomes and patient-reported measures can be progressively incorporated. In this way, the framework can function as a scalable implementation guide, allowing institutions with varying levels of digital infrastructure to begin operationalizing value-based care principles within outpatient pathways.

## Constraints and limitations

This paper is a case study and is therefore largely descriptive in nature. Its primary aim is to document and reflect on local experience in implementing VBC for outpatient conditions, rather than to evaluate effectiveness or establish causal relationships between interventions and outcomes. As such, the findings should be interpreted as implementation insights rather than evidence of clinical or economic impact. In addition, while improvement actions were identified and, in some cases, initiated following baseline analyses, long-term post-intervention outcome data were not available at the time of writing. The absence of longitudinal follow-up limits the ability to assess whether the proposed or implemented changes resulted in sustained improvements in outcomes, resource utilization, or patient experience. Future work will be required to evaluate the downstream impact of these improvement initiatives.

Data and measurement limitations were inherent to the outpatient settings examined. PROMs were at an early stage of implementation within the institution, and routine collection workflows were not yet established for either case study at the time of baseline assessment. As a result, PROMs could not be included in the baseline analyses and were instead introduced prospectively as part of the value-based care implementation process. For several indicators, ideal outcome definitions could not be operationalized using routinely available data, necessitating reliance on pragmatic proxies. In the radioiodine therapy case, biochemical treatment success could not be reliably determined from laboratory values alone due to active medication titration, leading to the use of levothyroxine initiation as a proxy outcome. While this approach was informed by literature review and validated through clinical record review and clinician engagement, it introduces a degree of imprecision that may affect comparability across settings.

The implementation framework and case studies are situated within the specific governance and financing arrangements of Singapore's public healthcare system. Institutional structures, data infrastructure, and national value-driven care programmes shaped both the selection of outcomes and the implementation process. As a result, certain elements of the framework may not be directly transferable to health systems operating under different funding or governance models.

Nevertheless, while contextual factors may limit direct generalizability, the underlying principles of structured scoping, iterative data exploration, and clinician-engaged improvement planning are likely to be applicable across a range of healthcare settings.

## Conclusion

This community case study describes a pragmatic framework for implementing value-based care for outpatient conditions within a large Asian public healthcare system and illustrates its application through two contrasting case studies. The SGH Value-Based Care Implementation Framework enabled systematic outcome selection, structured clinician–administrator engagement, and identification of actionable improvement opportunities beyond procedural care. By documenting local implementation experience, this study contributes practical insights to the limited literature on outpatient value-based care, particularly from Asian health systems. As healthcare systems face growing sustainability pressures driven by chronic disease and aging populations, sharing and adapting such implementation approaches may support more effective and scalable value-based transformation across diverse care settings.

## Data Availability

The original contributions presented in the study are included in the article/supplementary material, further inquiries can be directed to the corresponding author/s.
